# Poly(ADP-ribose) Polyremase-1 (PARP-1) Inhibition: A Promising Therapeutic Strategy for ETS-Expressing Tumours

**DOI:** 10.3390/ijms241713454

**Published:** 2023-08-30

**Authors:** Arnaud J. Legrand, Souhaila Choul-li, Vincent Villeret, Marc Aumercier

**Affiliations:** 1 CNRS, EMR9002 Integrative Structural Biology, F-59000 Lille, France; arnaud.legrand@icr.ac.uk (A.J.L.); vincent.villeret@univ-lille.fr (V.V.); 2 Univ. Lille, Inserm, CHU Lille, Institut Pasteur de Lille, U1167-RID-AGE-Risk Factors and Molecular Deter-minants of Aging-Related Diseases, F-59000 Lille, France; 3 Département de Biologie, Faculté des Sciences, Université Chouaib Doukkali, BP-20, El Jadida 24000, Morocco; choulli.s@ucd.ac.ma

**Keywords:** ETS transcription factors, PARP-1, pharmacological inhibition, cancer therapy, DNA damage

## Abstract

ETS transcription factors are a highly conserved family of proteins involved in the progression of many cancers, such as breast and prostate carcinomas, Ewing’s sarcoma, and leukaemias. This significant involvement can be explained by their roles at all stages of carcinogenesis progression. Generally, their expression in tumours is associated with a poor prognosis and an aggressive phenotype. Until now, no efficient therapeutic strategy had emerged to specifically target ETS-expressing tumours. Nevertheless, there is evidence that pharmacological inhibition of poly(ADP-ribose) polymerase-1 (PARP-1), a key DNA repair enzyme, specifically sensitises ETS-expressing cancer cells to DNA damage and limits tumour progression by leading some of the cancer cells to death. These effects result from a strong interplay between ETS transcription factors and the PARP-1 enzyme. This review summarises the existing knowledge of this molecular interaction and discusses the promising therapeutic applications.

## 1. Introduction

ETS transcription factors are a family of proteins encoded by a group of genes conserved in the evolution from metazoan to humans [1,2]. To date, 28 members of this family, divided into 12 groups, have been described in vertebrates [3]. These transcription factors are characterised by a well-conserved winged helix-turn-helix DNA-binding domain (DBD) that recognises specific DNA elements with a central 5′-GGA(A/T)-3′ core, called ETS-binding sites (EBS), found in the promoters of target genes. Even though all ETS family members share the same DBD, each ETS transcription factor has its DNA-binding properties that are tightly controlled to ensure a specific biological action. Concretely, DNA-binding properties of ETS transcription factors can be differentiated from each other by (i) slight variation in the recognition of EBS sequences [4], (ii) specific interactions with diverse binding partners, or (iii) differential post-translational modifications that modulate their affinity for DNA [3]. Nevertheless, ETS transcription factors are extensively co-expressed in many cell types (e.g., hematopoietic cells, breast and prostate tissues) and the biological specificity of each factor in these cells remains unclear [3].

In physiological processes, ETS transcription factors are mainly involved in embryonic development where they control cell growth, differentiation, migration, and apoptosis. These roles enable the successful conduct of many processes in the embryo such as morphogenesis, haematopoiesis, and angiogenesis [5]. However, the expression of ETS proteins is tightly controlled in adult tissues, and their neo- or over-expression is mainly associated with cellular transformation and cancer progression [6,7]. ETS transcription factors are involved in many carcinomas and leukaemias in humans. Moreover, they are often considered as markers of poor prognosis in these diseases [6].

Given their implications in cancer, various strategies have been employed to specifically target ETS proteins activity in tumours. Amongst these, studies investigated strategies using siRNA [8], splice-switching oligonucleotides [9], artificial or natural dominant-negatives [10,11], peptidomimetic inhibitors [12], or small molecules that bind to the EBS [13]. Another widely used strategy developed small molecules that inhibit ETS proteins activity by (i) changing their localization in the nucleus [14,15], (ii) blocking their interaction with binding partners [16,17], (iii) inhibiting their transcriptional activity [18,19,20,21,22,23,24] or (iv) repressing their expression [25]. Until now, only two clinical trials were performed in patients with Ewing’s sarcoma using inhibitors of the ETS fusion protein, EWS-Fli1. The first trial using mithramycin has unfortunately failed [26] and the second one using TK-216 is under evaluation in phase I (NCT02657005). 

However, ground-breaking findings have identified poly(ADP-ribose) polymerase-1 (PARP-1), a key DNA repair enzyme, as a direct binding partner of ETS proteins Erg, Fli1, and Ets-1. Furthermore, these studies demonstrated that pharmacological inhibition of PARP-1 specifically sensitises ETS-expressing cancer cells to DNA damage and limits tumour progression [27,28,29]. These findings are especially interesting, since PARP-1 inhibitors (PARPi) are already used in clinical trials and showed good efficiency, notably in ovarian and breast cancers where ETS proteins are often overexpressed [6,30,31,32]. This review reports the strong interplay between ETS transcription factors and PARP-1 enzyme and discusses the promising therapeutic applications.

## 2. ETS Transcription Factors Expression in Cancers

### 2.1. Expression and Involvement in Cancers

**Figure 1:**
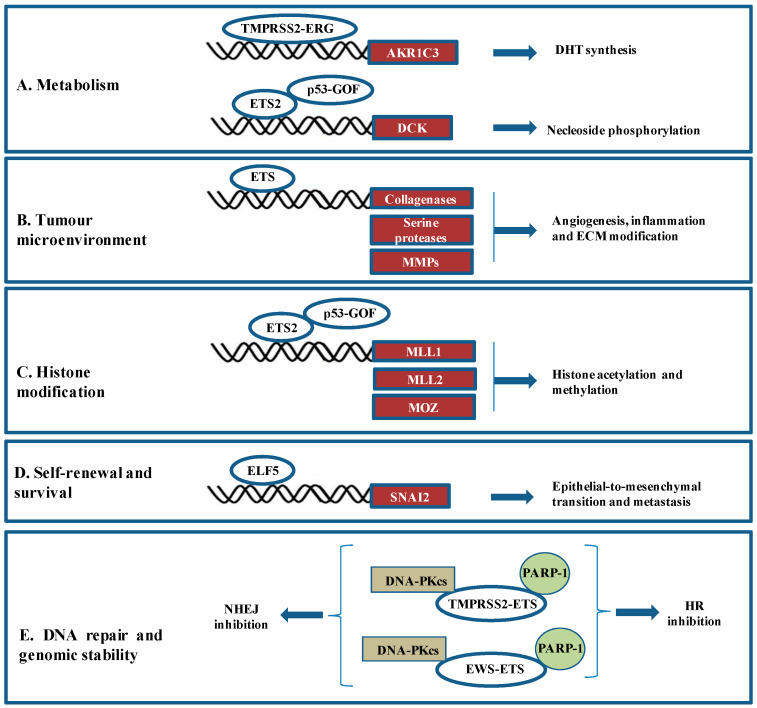
Mechanisms driven by ETS factors in solid tumours. (**A**) TMPRSS2–ERG activates the expression of AKR1C, the androgen biosynthesis enzyme. ETS2 complexed to the p53-GOF mutant regulates the transcription of DCK, an enzyme that phosphorylates deoxyribonucleosides. (**B**) ETS factors modulate the expression of numerous factors (e.g., collagenases, serine proteases and MMPs) implicated in angiogenesis, inflammation, and ECM modification. (**C**) Synergistic cooperation between ETS2 and p53-GOF mutants to activate the transcription of MLL1, MLL2 and MOZ, enzymes of histone modifications. (**D**) ELF5, an ETS transcription factor represses the expression of SNAI2, an inducer of EMT and metastasis. (**E**) ETS fusion proteins (TMPRSS2-ERG and EWS-FLI1) interact with PARP-1 and DNA-PKcs. PARP-1 sequestration prevents DNA repair by HR and DNA-PKcs sequestration inhibits DNA repair by NHEJ.

Since their discovery in 1983 as part of the gag-myb-ets transforming fusion protein of an avian replication-defective retrovirus (E26), ETS transcription factors have been associated with carcinogenesis [33,34]. Indeed, the original ETS family member v-ets, a homolog of Ets-1, can transform fibroblasts, myeloblasts, and erythroblasts and cause mixed erythroid–myeloid and lymphoid leukaemia in chicken [35]. Thereafter, a correlation between ETS genes expression level and tumour progression has been established in a wide range of human neoplasias such as thyroid, pancreas, ovarian, liver, colorectal, or lung carcinomas and a great number of studies showed the complex and essential function of ETS proteins in the progression and prognosis of breast and prostate carcinomas, and Ewing’s sarcoma, as well as in diverse leukaemias [3,6]. This extensive involvement can be explained by their roles at all stages of carcinogenesis processes. Indeed, ETS transcription factors promote transformation, invasion, angiogenesis, and inflammation, as well as metastasis through a wide range of molecular and cellular mechanisms including metabolism, tumour microenvironment, histone modifications, cancer self-renewal and survival and DNA repair (reviewed in more detail in [6,36] and summarized in Figure 1). Indeed, ETS factors have an impact on the metabolism of steroids and nucleotides, which are necessary for the survival of tumour cells. TMPRSS2-ERG modulates the expression of AKR1C3, the androgen biosynthesis enzyme, decreasing dihydrotestosterone (DHT) synthesis [37]. ETS2 and p53-GOF activate together the expression of deoxycytidine kinase (DCK), an enzyme that phosphorylates deoxyribonucleosides [38] (Figure 1A). ETS factors modulate angiogenesis, inflammation, and extracellular matrix (ECM) modification through the direct transcriptional regulation of collagenases, serine proteases, and matrix metalloproteinases (MMPs) [39] (Figure 1B). Modulating chromatin dynamics and epigenetics is a third molecular mechanism through which ETS factors mediate tumorigenesis. Indeed, ETS2 cooperates with p53-GOF mutants to regulate the histone acetyltransferase MOZ, the histone methyltransferases mixed-lineage leukaemia 1 (MLL1), and MLL2. Analysis of The Cancer Genome Atlas demonstrated high expression of MLL1, MLL2, and MOZ in p53-GOF patient-derived tumours [40] (Figure 1C). ETS factors are also involved in cancer self-renewal and survival by inhibiting the epithelial–mesenchymal transition (EMT) in mammary gland development and breast cancer metastasis. Elf5, an ETS transcription factor, represses the transcription of Snail2, an inducer of EMT [41] (Figure 1D). The modulation of DNA repair is the latest molecular mechanism by which ETS factors regulate tumorigenesis (Figure 1E). The ETS fusion protein, TMPRSS2-ERG is an interaction partner of PARP-1 and DNA-dependent protein kinase catalytic subunit (DNA-PKcs) [28]. PARP-1 sequestration prevents DNA repair by homologous recombination (HR). TMPRSS2-ERG-PARP-1 interaction inhibits the phosphorylation of DNA-PKcs, thus blocking DNA repair by the non-homologous end joining (NHEJ) [42]. Moreover, EWS-FLI1 and EWS-ERG directly interact with PARP-1 and DNA-PKcs, blocking DNA repair in Ewing sarcoma and prostate cancer [27].

It is possible to differentiate several groups of ETS transcription factors regarding their specific role in particular types of neoplasia. A report by Wei et al. demonstrated that we can separate ETS proteins from each other by analysing their DNA-binding profiles and their affinity for slight base variation within the EBS (Table 1) [4]. 

This study revealed that the ETS-binding profiles cluster into four distinct classes and that most of the ETS transcription factors involved in solid tumours (e.g., ETS, PEA3, and ERG groups; Table 1) were found in the class I and bind either a 5′-GGAA-3′ or a 5′-GGAT-3′ consensus core [4]. On the contrary, ETS proteins that are known to be mainly involved in leukaemias are clustered in classes III and strictly recognise a 5′-GGAA-3′ core (Table 1). In class II, clustered members preferentially recognise a 5′-GGAA-3′ core and are involved in both solid tumours and leukaemias (Table 1). Finally, the one member only of class IV, Spdef, which binds a 5′-GGAT-3′ core [4], is known on the one hand to be involved in tumour progression and endocrine resistance in breast cancers [43], and on the other hand to act as a putative tumour suppressor in prostate and colorectal carcinomas [44,45]. Thus, we could hypothesise that the ability to recognise a 5′-GGAT-3′ core might be linked to the involvement in solid tumour progression by selecting specific target genes.

**Table 1:** ETS transcription factors family members, their associated cancers, and their interaction with PARP-1.

Class [4]	Group [4]	Members	Associated Cancers [3,6]	Interaction with PARP-1 *
I (GGAA/T)	ETS	Ets-1 Ets-2	Breast, ovarian, lung, colorectal, thyroid, uterus, melanoma, gastric, prostate, pancreas, liver, leukaemias…	Yes, directly (Ets-1) [29]
	ERG	Erg Fli1 Fev	Prostate, Ewing’s Sarcoma, ovarian…	Yes, directly (Erg) and in complex (Fli1) [27,28]
	PEA3	Etv1 Etv4 Etv5	Breast, Ewing’s sarcoma, lung, gastric, prostate…	Yes, in complex (Etv1) [28]
	TCF	Elk1 Elk3 Elk4	Prostate	Yes, in complex (Elk1) [46]
	ERF	Erf Etv3 Etv3L		
	ER71	Etv2	Breast	
	GABP	GabpA		
II (GGAA/t)	ESE	Elf3 Ehf Elf5		Yes, in complex (Elf3) [47]
	TEL	Etv6 Etv7	Leukaemias	
	ELF	Elf1 Elf2 Elf4	Prostate, endometrial, ovarian…	
III (GGAA)	SPI	Spi1 SpiB SpiC	Leukaemias	Yes, in complex (Spi1) [48]
IV (GGAT)	PDEF	Spdef	Prostate, breast	

* Direct interactions have been demonstrated by in vitro assays using recombinant proteins, whereas in complex refers to interaction identified by co-immunoprecipitation.

### 2.2. ETS Fusions and Cancers

Expression or deregulation of ETS transcription factors in cancer cells is mostly due to the activation of ETS genes by amplifications or punctual mutations, but also by chromosomal translocations. In this case, ETS genes are often involved in chromosomal translocations which result in a fusion with other proteins [6]. There are different types of ETS fusions. The most studied are ETS gene fusions in Ewing’s sarcoma and prostate cancer [49,50,51]. Other fusions involve Etv6 and several proteins in a wide range of leukaemias, but we will not approach them here (reviewed in [52]).

Ewing’s sarcoma is a rare cancer affecting bones and soft tissues occurring in teenagers and young adults. Cytogenetically, this cancer is defined by its signature translocations that produce fusion proteins with strong carcinogenic potential. In 90% of cases, the fusion occurs between the encoding regions of the N-terminal portion of EWS, an RNA binding protein, and the C-terminal portion of Fli1, an ETS transcription factor. In the remaining 10%, the fusion occurs between EWS and other ETS factors from the same group, Erg [49]. 

The resulting fusion proteins act as transcription factors and regulate a wide range of genes involved in proliferation, carcinogenesis, and tumour progression [49,53]. EWS-ETS fusions are considered as the keystone of Ewing’s sarcoma development. Thus, their targeting is a high priority to treat this disease [49,53].

In prostate cancer, numerous gene fusions are found involving different ETS transcription factors such as Erg, Etv1, Etv4, and Etv5 (Table 1) [50]. However, none of them gained as much importance in clinical practice as the rearrangement involving TMPRSS2 and Erg genes. This rearrangement, found in approximately 50% of prostate cancers, results in a gene fusion, TMPRSS2:Erg, which places ERG expression under the transcriptional control of androgen and oestrogen receptors [54]. TMPRSS2:Erg fusion might be associated with the transition to invasive cancer by over-regulating Erg target genes involved in migration, invasion, and metastasis [50,55,56]. That is why this rearrangement is considered both as a diagnosis tool and a potential therapeutic target in aggressive prostate cancer [50,56]. 

## 3. PARP-1 Inhibition in Cancer Therapy

### 3.1. The Plethoric Roles of PARP-1 in Cancer Cells

PARP-1, one of the most abundant proteins in the cell nucleus, is the founding member of the PARP family of enzymes. Its catalytic activity is characterised by the addition of ADP-ribose polymers, by consuming cellular NAD^+^, on the target proteins with which PARP-1 interacts. This post-translational modification is called poly(ADP-ribosyl)ation (PARylation) [57]. Since its discovery in 1963, PARP-1 has mainly been associated with DNA repair processes, notably in the Base Excision Repair (BER) mechanism where PARP-1 facilitates the association and dissociation of the repair complexes after recognition of single strand breaks (SSBs). Furthermore, PARP-1 is also involved as a backup enzyme for the repair of diverse single-strand and double-strand DNA lesions like in HR, NHEJ, and Nucleotide Excision Repair (NER) [58,59].

In a sense, PARP-1 is situated at the crossroads of signalisation pathways involved in the sensing of genotoxic, metabolic, and oncogenic stresses. Thus, its function could be described as a sensor of the cellular stresses, which explains its plethoric roles in the cell [60]. Moreover, PARP-1 is integrated in the structure of chromatin. This localisation also allows PARP-1 to be involved in transcription processes since this enzyme controls chromatin remodelling and interacts with transcription factors on the promoter of many genes [61]. It has been argued that PARP-1 could be involved in the regulation of 3.5% of all genomes in embryonic cells. Even if it is very difficult to know if this effect is due to transcription regulation or chromatin structure defects, it shows the importance of PARP-1 in transcription processes [62].

Finally, PARP-1 catalytic activity assures the control of energetic resources, since this activity consumes the stock of NAD^+^. Therefore, only cells with a high metabolism, like cancer cells, can maintain a sustained PARylation activity at the risk of depleting all NAD^+^, which could lead to cell death [60].

### 3.2. PARP-1 Inhibition in Cancer Cells and Clinical Trials

First, PARP-1 was considered as a tumour suppressor due to its plethoric roles in DNA repair. It was argued that suppression of its expression and/or its activity could be involved in cancer development. However, transgenic mice deficient in PARP-1 expression do not show any spontaneous tumour development, even though they are more sensitive to alkylating agents which provoke in these mice liver and colorectal cancers with a greater frequency [57,63]. 

Indeed, once the tumour is formed, PARP-1 expression tends to be increased. PARP-1 expression is higher in breast [64,65], liver [66], colorectal carcinomas [67], and melanomas [68]. Furthermore, studies showed that the PARylation level is increased in cancer tissues [69,70]. We may suppose that tumour cells can hijack PARP-1 activity to promote DNA repair, and therefore cancer progression, without activating cell death pathways which are often defective in these cancers. 

Given the major role of PARP-1 in cancer cells, many pharmacological inhibitors have been designed to set up targeted strategies in the hope of preventing effective DNA repair in these cells. The first approach is to improve chemo- and radiotherapy efficacy by combining them with PARPi. This has led to numerous clinical trials with diverse combinations of PARPi with platinum drugs (cisplatin or carboplatin), alkylating agents (temozolomide), doxorubicin, topoisomerase I inhibitors (topotecan), antimetabolites (gemcitabine or capecitabine), paclitaxel, eribulin or vinorelbine. PARP inhibitor combination therapy has been reviewed in more detail in [71,72]. A second approach is to use PARPi as a single agent on cancer cells with a particular genetic background. The initial strategy is the use of PARPi in BRCA1/2-deficient cells. In the S phase, cells can only repair double-strand breaks (DSBs) occurring during DNA replication by going through HR, a process where BRCA1/2 is essential. BRCA1/2-deficient cells are deficient in HR and need PARP-1 to prevent DSBs formation during replication. Therefore, PARP-1 inhibition in these cells leads to the massive formation and accumulation of unrepaired DSBs and, eventually, cell death [73,74]. This strategy, which follows the principle of synthetic lethality, is applied in clinical trials and is effective in BRACA1/2-deficient tumours [75,76] and then extended to many other HR proteins deficiencies, such as deficiency in ATM and Rad51 [77]; for a list of clinical trials on PARP inhibitors, see Table 2.

### 3.3. Limitation of PARP-1 Inhibitors in Cancer Therapy

The different phases of clinical trials using PARPi have given encouraging results. Nevertheless, resistance to PARPi is developed in cancer cells and clinical trials through several mechanisms (reviewed in [91,92]). The first mechanism of PARPi resistance is the upregulation of drug-efflux transporters such as the ATP-binding cassette transporter, ABCB1, which prevents the intracellular accumulation of PARPi [93,94]. Mutations in PARP-1 that reduce the binding affinity of PARPi to the catalytic domain of PARP-1 or reduce the affinity of PARP-1 to DNA also produce resistance to PARPi [95,96].

Cancer cell resistance to PARPi might also occur through restoration of HR upon reactivation of BRCA1/2 function or loss of DNA end protection. BRCA1/2 function is restored through reversion mutations [97] or epigenetic modifications [98,99]. Loss of DNA end protection occurs only in BRCA1-deficient cells upon loss of 53BP1 [94,100], an NHEJ factor, and the downstream factors such as REV7, RIF1, and the shieldin complex [101,102,103].

Another mechanism of PARPi resistance is the restoration of replication fork stability by loss of PTIP, EZH2, or RADX expression [104,105,106] or loss of cell-cycle checkpoint arrest in BRCA1/2-deficient cells [107].

PARPi resistance is circumvented by improving PARPi sensitivity through its combination with other inhibitors of proteins involved in various cellular mechanisms such as inhibitors of ATR [108], WEE1 [109,110], PD-L1 [111], or c-Met [112].

Certain PARPi showed some weaknesses in clinical trials and some studies have underlined the disparity of effectiveness among inhibitors. Moreover, it seems as if there is a lack of specificity to target cancer cells versus normal cells unless the tumours are sensitised to PARP-1 inhibition because of deficiencies in DNA repair or specific genetic background [60]. For this reason, the greatest remaining challenge for the use of PARPi in clinics is finding strong biomarkers that would indicate whether cancer cells would be responsive to this treatment. In this spirit, recent findings demonstrated that biomarkers of sensitivity for PARP-1 inhibition are not always deficiencies but could also be gains of expression, and are not always directly linked to DNA repair. This is the case with ETS proteins expression. 

## 4. Molecular Interplay between ETS Transcription Factors and PARP-1 Enzyme

### 4.1. Regulation of PARP-1 Expression and Activity by ETS Transcription Factors

The interplay between PARP-1 and ETS proteins starts with the regulation of the PARP1 gene upon cellular stresses. Indeed, in Ewing’s sarcoma cells, analysis of the PARP1 promoter showed its up-regulation in response to DNA damage, and this is carried out by ETS transcription factors, Ets-1 and Fli1 [113,114]. Furthermore, the depletion of Fli1 in Ewing’s sarcoma cells leads to the disappearance of PARP-1 expression [27]. This control of PARP-1 expression allows Ewing’s sarcoma cells to resist ionizing radiation and genotoxic agents [113,114]. Another study showed that in ovarian cancer cells, Ets-1 activates PARP-1 expression synergistically with histone modification H3K9 by binding to the hypomethylated EBS present in the PARP1 promoter [115]. To keep the balance of the NAD^+^ stock, ETS transcription factors also up-regulate the expression of the poly(ADP-ribose) glycohydrolase (PARG) enzyme which degrades PAR to reform the NAD^+^ level [116]. Thus, ETS transcription factors promote the global process of PARylation/dePARylation and therefore increase the efficiency of DNA repair in cancer cells. 

However, the interplay between PARP-1 and ETS factors is not limited to the control of PARP-1 expression. Indeed, work by ourselves and others demonstrated protein–protein interaction between PARP-1 and ETS family members, Ets-1, and Erg (WT and fusion) [28,29]. This interaction is triggered by direct contact between PARP-1 and the ETS domain common to all ETS factors [117]. So, we might assume that PARP-1 could interact with all ETS proteins, a supposition supported by the fact that PARP-1 is found in immunoprecipitated complexes with several ETS members such as Fli1 [27] and Spi1 [48]. 

In the case of Ets-1, this interaction showed interesting features regarding PARP-1 activity. Indeed, the C-terminal portion of Ets-1, containing the ETS domain, can promote PARylation activity in vitro in a DNA-independent manner by interacting with PARP-1 [29]. Therefore, the direct interaction between PARP-1 and ETS transcription factors could promote PARylation in cells. This is supported by the fact that Ets-1 depletion in MDA-MB-231 cells, a highly invasive breast cancer cell line, drastically decreases the PARylation level and the ability of these cells to overcome genotoxic stress [118]. Furthermore, a report showed that Erg overexpression confers radiation resistance to prostatic cancer cell lines and that this is mainly through the increase in the PARylation level [119].

Thus, ETS transcription factors promote both PARP-1 expression and activity to allow cancer cells to improve the efficiency of DNA repair and overcome genotoxic stress. 

### 4.2. Control of ETS Transcription Factors Functions by PARP-1

Since it has been reported by Cohen-Armon et al., it has been known that PARP-1 could positively regulate ETS factors transcriptional activity, in this case, Elk-1 factors activity, by promoting their phosphorylation by ERK-2 [46]. Given the fact that PARP-1 is known to have numerous roles as a transcription cofactor, we and others have tried to find out whether PARP-1 could directly regulate transcriptional activity of the ETS factors on target gene promoters. The results showed that PARP-1 depletion leads mainly to a decrease in ETS factors transcription activity [28,29]. This would tend to prove that PARP-1 is an essential component of transcription platforms, as has already been observed for other transcription factors.

Nevertheless, although PARP-1 is needed as an interaction partner, its catalytic activity is not always necessary to ensure transcription. Furthermore, a possible role of PARylation is to promote a proper dissociation of protein–protein interactions to avoid blockade in the processes and ensure the dynamism and renewal of the complexes [57,61]. PARylation of the binding partner could then be a determinant factor in the role of PARP-1 in transcription. In the case of Erg, PARP-1 depletion and inhibition both gave the same decrease in Erg transcriptional activity, but there is no evidence of Erg PARylation [28,119]. On the contrary, in the case of Ets-1, PARP-1 knock-out provoked a decrease in Ets-1 transcriptional activity, whereas PARP-1 inhibition caused an increase [29]. This could be explained by the fact that Ets-1 is PARylated and that this PARylation is needed to dissociate Ets-1 from the promoter.

In addition, we demonstrated that PARP-1 inhibition caused a strong accumulation of Ets-1 in cancer cells [29]. To understand the underlying mechanism, we used an Ets-1 isoform which is not sensitive to proteasomal degradation and showed that this isoform was not accumulated during PARP-1 inhibition [29]. Thus, the PARylation of Ets-1 might be involved in its degradation by the proteasome. It is known that the PARylation of proteins could promote the recruitment of E3 ubiquitin ligases, such as RNF146 or CHFR, which target the PARylated proteins for proteasomal degradation [120]. Thus, PARP-1 might regulate the protein level of ETS factors with the only condition that they are PARylated.

## 5. Cellular Consequences of PARP-1 Inhibition on ETS-Expressing Tumour Cells

### 5.1. PARP-1 Inhibition Slows down ETS-Expressing Tumour Growth by Inhibiting Invasion and Metastasis and Decreasing Cell Survival

Given the involvement of PARP-1 on ETS-driven transcription, studies have tried to elucidate whether PARP-1 inhibition could counteract ETS factors’ role in tumour growth and progression. In vitro invasion assays showed that PARP-1 inhibition attenuates Erg- and Etv1-dependent invasion in prostatic cancer cell lines [28]. The same observations were made for Ewing’s sarcoma cells expressing EWS-Fli1 [27]. Furthermore, PARP-1 inhibition leads to a significant decrease in the intravasation capacity of Erg-expressing cancer cells, which is more proof of the reduced ability of ETS-expressing cells to commit invasion under PARP-1 inhibition. But, very surprisingly, the authors did not notice any effect on the proliferation and survival of Erg-expressing cells [28]. However, another study observed that the presence of Erg gene fusion facilitates the activation of senescence when PARP-1 inhibition is combined with low-dose-rate radiation [121]. Furthermore, in the report that identified EWS-Fli1 expression as a strong biomarker of sensitivity to PARP-1 inhibitor, Garnett et al. showed that most of EWS-Fli1-expressing cells undergo apoptosis after only 3 days of treatment [122]. Additionally, we and others observed that PARP-1 inhibition leads Ets-1-expressing cells to death even if Ets-1 activity is increased, in this case as shown by up-regulation of MMP-3 and under-regulation of BRCA1 [29,118]. Altogether, these results tend to prove that PARP-1 inhibition decreases pro-oncogenic activities of ETS transcription factors and cell survival, even if the precise mechanism seems to differ across ETS members and cell lines. 

Finally, works by Brenner et al. demonstrated that pharmacological inhibition of PARP-1 inhibits Erg- and EWS-Fli1 positive, but not negative, tumour xenograft growth in mice and the ability of these tumours to form metastasis. These effects are even more striking if PARP-1 inhibition is combined with chemotherapeutics such as temozolomide [27,28]. 

### 5.2. PARP-1 Inhibition Causes the Accumulation of Unrepaired DSB in ETS-Expressing Cells

Although the decrease in invasion and metastasis after PARP-1 inhibition could easily be explained by disruption of ETS-driven transcriptional activity, the impact on tumour growth and cell survival is more surprising. Indeed, how can we explain that ETS-positive cells are more sensitive to PARP-1 inhibition than ETS-negative cells, when we could have expected that PARP-1 inhibition would just have neutralised ETS factors’ oncogenic effects? This supposes deleterious effects of ETS expression.

It is known that ETS transcription factors cause genomic instability, even if the mechanism is poorly understood [123]. Indeed, overexpression of ETS factors in cancer cells causes the formation of numerous DSBs [28,119]. Yet, PARP-1 inhibition impressively increases this ETS-dependent formation of DSB in cancer cells. This increase has been observed for treated cells expressing Ets-1, Erg (WT and fusion), and EWS-Fli1 by evaluating the level of γH2AX, 53BP1, and Rad51 foci, which are well-known DSB markers, and by neutral comet assay [27,28,29,119]. Of course, the increase in DSB in these cells is dependent on the expression of ETS factors, since ETS depletion abrogates DSB formation during PARP-1 inhibition.

Thus, it seems as if ETS-dependent sensitivity to PARP-1 inhibition is mainly due to an increase in DSB which is highly toxic for the cells. However, the cause of this increase is still under investigation.

## 6. Remaining Questions and Therapeutic Applications

### 6.1. PARP-1 and ETS Transcription Factors Interplay: Controlling DNA Damage

An increasing amount of studies have now reported that PARP-1 inhibition causes an important increase in DSB DNA damage in cancer cells expressing ETS transcription factors such as Erg, Fli1, or Ets-1. Yet, although this observation is recurrent across publications, there is no convincing mechanistic explanation for this phenomenon. Do ETS transcription factors provoke DSB directly or indirectly? Indeed, ETS factors overexpression has been shown to cause the accumulation of DSB in cancer cells. However, we do not know if these lesions are DSBs from the start or unrepaired SSBs, caused, for example, by oxidative attacks, converted into DSBs during DNA replication. Furthermore, there is no demonstration of a direct link between DSB formation and ETS factors’ transcriptional and/or other functional activities. 

**Figure 2:**
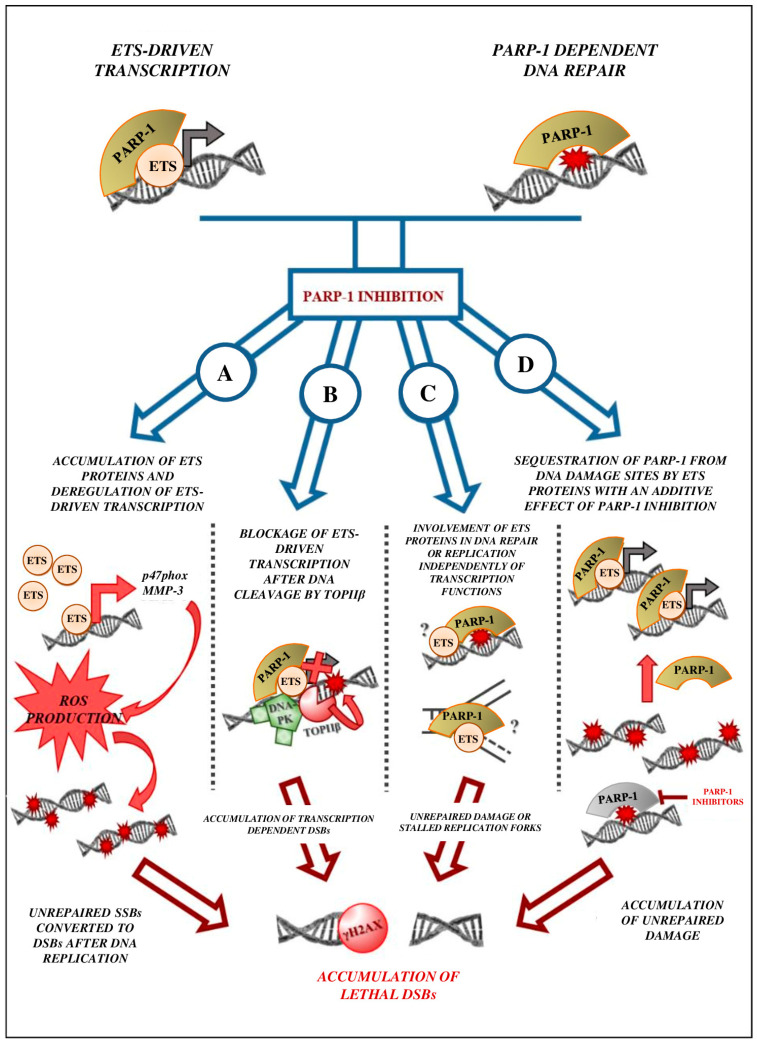
Different hypotheses of ETS-driven DSB formation upon PARP-1 inhibition. (**A**) Ets-1 model: PARP-1 inhibition causes an accumulation of Ets-1 and an increase in its transcriptional activity. This provokes the production of ROS and inhibition of DNA repair, and therefore unrepaired SSBs are converted into DSBs after replication. (**B**) Erg model: PARP-1 inhibition blocks ETS-driven transcription after cleavage of DNA by topoisomerase IIβ. (**C**) Non-transcriptional (Myc) model: ETS factors have a non-transcriptional function in DNA damage during DNA replication; PARP-1 inhibition leads to defects in this process. (**D**) Sequestration model: ETS proteins sequestrate PARP-1 from DNA damage sites, decreasing DNA repair efficiency even more if PARP-1 is inhibited. All these models lead to an accumulation of lethal DSBs in cancer cells.

What could be the mechanism(s) of the ETS-driven formation of DSB, then? The first hypothesis would be that ETS-dependent formation of DSB is the consequence of ETS-driven transcriptional activity (Figure 2A). Indeed, on the one hand, Ets-1, for example, is known to activate reactive oxygen species (ROS) production by enhancing the expression of components of the nicotinamide adenine dinucleotide phosphate (NADPH) oxidase complex, such as p47^phox^ [124]. Furthermore, Ets-1 also increases indirectly ROS production by transactivating target genes such as MMP3 [125,126], an enzyme known to increase NADPH oxidase complex activity [127]. On the other hand, Ets-1 indirectly inhibits DNA repair by repressing BRCA1 expression, but also by forcing cell proliferation, through up-regulation of cyclin D1 and E and CDK2, which gives less time to the cell to repair its DNA [126,128]. This hypothesis is consistent with the fact that Ets-1 is accumulated during PARP-1 inhibition and is transcriptionally active [29]. Moreover, this also explains the increase in genotoxic stress when ETS factors are overexpressed.

Nevertheless, reports by Brenner et al. showed that Erg and EWS-Fli1 transcriptional activity is decreased by PARP-1 inhibition on a set of several Erg target genes [27,28]. Such a difference between Ets-1 and the ERG group could be explained by the fact that Ets-1 is PARylated and that this PARylation controls its protein level. Yet, overexpression of Erg and Fli1 also causes a significant amount of DSBs, which are increased during PARP-1 inhibition [27,28]. The authors suppose that the PARP-1 function on ETS transcription activity could be similar to its function in transcription processes mediated by nuclear receptors (NR) such as androgen and oestrogen receptors (Figure 2B) [28]. Indeed, NR-driven transcription is known to necessitate DSB formation by topoisomerase IIβ to decompact chromatin. These DSBs are repaired with the help of PARP-1 and DNA-PK. PARP-1 inhibition blocks NR-driven transcription; however, there is no evidence that the DSBs remain unrepaired [129]. The existence of a similar mechanism for ETS-driven transcription, as far as we know, is yet to be investigated.

A third possibility is that the ETS-driven formation of DBS could be completely independent of their transcriptional activity. Indeed, some transcription factors are known to cause DNA damage independently of transcription processes. For example, Myc is known to cause DNA damage, and this is, at least partially, because of a non-transcriptional role in DNA replication [130]. A similar mechanism for ETS factors might be conceivable (Figure 2C). Finally, taking into account the strong physical interaction between ETS factors and PARP-1, we could imagine that overexpression of ETS proteins causes a limited but effective sequestration of PARP-1 because of these transcription factors. Therefore, this could decrease the efficiency of DNA repair. PARylation inhibition would then have an additive effect by finishing the suppression of any PARP-1 activity on DNA damage sites (Figure 2D). 

### 6.2. Extending the Concept

Until now, PARP-1 was shown to physically interact with Ets-1 and Erg. However, the strong functional interaction with Fli1 and the fact that PARP-1 interacts with the ETS domain might lead us to think that PARP-1 directly interacts with all ETS transcription factors. Indeed, PARP-1 is functionally linked to a major part of class I ETS factors, but also with members of class II and III (Table 1). Extending the concept to all ETS transcription factors is far from anecdotal. For the moment, PARP-1 inhibition shows strong effects on ETS-expressing cells from prostatic, breast carcinomas and Ewing’s sarcoma cell lines. Extending the concept would not only spread the use of PARPi over the wide range of carcinomas involving Ets-1 or Erg expression, but also over numerous leukaemias (Table 1). The potential therapeutic applications would be extensive.

### 6.3. Using PARPi to Target ETS-Expressing Tumours

Studies have reported that ETS expression sensitises cancer cells to PARP-1 inhibition as much as or more than BRCA1/2 deficiency [27,28,122]. These data lead us to think that PARPi might be used as a single agent to selectively kill ETS-expressing cells in tumours.

Nevertheless, the efficacy of such treatment would probably be greatly improved by combining PARP-1 inhibition with another therapy, as it appears that the use of a PARP-1 inhibitor, niraparib, as a single agent, was not sufficient to observe a correlation between ETS rearrangements and tumour response to treatment in a published clinical trial on sporadic cancers [131]. Indeed, it has been shown that combining PARPi and radiation, even at a low-dose rate, improves the sensitivity of Erg- and EWS-Fli1-expressing cells to the treatment by increasing the rate of senescent cells and even more by leading to cancer cell death [121,132]. In the same way, combining PARPi with chemotherapeutics such as temozolomide or doxorubicin greatly synergises the toxic effects of the treatment [27,29].

Moreover, it is also possible to combine PARP-1 inhibition with other therapies that target proteins related to ETS factors. For example, DNA-PK inhibitors greatly sensitise Ets-1-expressing cells to PARP-1 inhibition [118]. This can be explained by the fact that DNA-PK is an interaction partner of ETS transcription factors and a regulator of their activity [28,47,133]. The same effect might be observed for the inhibition of androgen biosynthesis in castration-resistant prostate cancer (CRPC) due to the interplay between the androgen receptor and TMPRSS2:Erg [54]. However, a phase II clinical trial studying the impact of combining androgen receptor inhibitor (abiraterone) with PARPi (veliparib) for patients with metastatic CRPC with or without ETS-rearrangement has demonstrated that ETS expression did not predict the effect of PARP-1/androgen receptor co-inhibition [134]. According to the authors, this result is due to the high prevalence of deficiency in DNA damage repair genes, which was not known at the time of conception of this study. 

## 7. Concluding Remarks

In conclusion, we and others have provided evidence that ETS transcription factors may be potential new candidates for biomarkers of cancer cell sensitivity to PARP-1 inhibition. Beyond prostate cancer and Ewing sarcoma, PARPi could be used to selectively kill ETS-expressing tumours in numerous cancers such as breast, lung, colorectal, or ovarian carcinomas and leukaemias. Time will tell if this therapeutic strategy is viable, but these preliminary results offer us the first actual strategy practicable in the clinic to target ETS-expressing cells, which is already a tremendous advance in this field.

## Figures and Tables

**Table 2:** Clinical trials with PARP inhibitors.

PARPi	Clinical Trial Phase	Cancer Type	Comments	Reference
Olaparib	I (NCT00516373)	Solid tumours with gBRCA mutations	First trial of Olaparib as a single agent	[76]
	II (NCT00679783)	TNBC with gBRCA mutations and high-grade ovarian cancer	No response in breast cancer	[30]
	II (NCT00753545)	Platinum-sensitive relapsed serous ovarian cancer with gBRCA mutations	This trial helped the FDA to approve the first PARP inhibitor in 2014	[78]
	III (NCT01874353; SOLO-2)	Platinum-sensitive relapsed ovarian cancer with BRCA mutations	This trial supported FDA approval	[79]
	III (NCT01844986; SOLO-1)	BRCA-mutated ovarian cancer after platinum-based chemotherapy	This trial supported FDA approval as first-line maintenance therapy in ovarian cancer in 2018	[80]
	III (NCT02000622)	gBRCA-mutated breast cancer	This trial supported FDA approval	[81]
	III (NCT02184195; POLO)	BRCA-mutated pancreatic cancer	This trial supported FDA approval as first-line maintenance therapy in pancreatic cancer	[82]
Rucaparib	I (not applicable)	Melanoma and solid tumours	First trial in combination with temozolomide	[83]
	I/II (NCT01482715; Study 10)	Solid tumours and BRCA-mutated ovarian cancer	This trial supported FDA approval	[84]
	II (NCT01891344; ARIEL2)	Platinum-sensitive high-grade ovarian cancer	This trial supported FDA approval and Foundation Medicine’s companion diagnostic	[85]
	III (NCT01968213; ARIEL3)	High-grade, recurrent, platinum-sensitive ovarian cancer	Supported FDA approval in 2018 for second-line therapy	[86,87]
Niraparib	II (NCT02354586; QUADRA)	Ovarian cancer	This trial supported FDA approval as maintenance therapy	[88]
	III (NCT01847274; ENGOT-OV16/NOVA)	Platinum-sensitive, recurrent ovarian cancer	This trial supported FDA approval as maintenance therapy	[89]
Talazoparib	III (NCT01945775; EMBRC)	Advanced breast cancer	This trial supported FDA approval	[90]

FDA: Food and Drug Administration; gBRCA: germline BRCA; TNBC: triple-negative breast cancer.

## Data Availability

Not applicable.

## Abbreviations

γH2AX	phosphorylated H2A histone member X
53BP1	tumour suppressor p53-binding protein 1
ADP	adenosine diphosphate
ATM	ataxia telangiectasia mutated
ATR	ataxia telangiectasia and Rad3 related
BER	base excision repair
BRCA1	breast cancer type 1 susceptibility protein
CDK2	cyclin-dependent kinase 2
CHFR	checkpoint with forkhead and RING finger domain protein
c-Met	mesenchymal-epithelial transition factor
CRPC	castration-resistant prostate cancer
DBD	DNA-binding domain
DCK	deoxycytidine kinase
DHT	dihydrotestosterone
DNA	deoxyribonucleic acid
DNA-PK	DNA-dependent protein kinase
DSB	double-strand break
EBS	ETS-binding site
ECM	extracellular matrix
Ehf	ETS homologous factor
ELF	E74-like factor
EMT	epithelial–mesenchymal transition
ER71	Ets-related protein 71
Erf	Ets-2 repressor factor
ERG	ETS-related gene
ERK-2	extracellular signal-regulated kinase-2
ESE	epithelium-specific Ets
ETS	E-twenty-six specific
Etv	ETS translocation variant
Fev	fifth Ewing variant
Fli1	friend leukaemia integration 1
GABP	GA-binding protein
HR	homologous recombination
MLL	methyltransferases mixed-lineage leukaemia
MMP	matrix metalloproteinase
MRN	Mre11-Rad50-Nbs1
NAD+	nicotinamide adenine dinucleotide
NADPH	nicotinamide adenine dinucleotide phosphate
NER	nucleotide excision repair
NHEJ	non homologous end joining
NR	nuclear receptors
PAR	poly(ADP-ribose) polymers
PARG	poly(ADP-ribose) glycohydrolase
PARP-1	poly(ADP-ribose) polymerase-1
PARPi	poly(ADP-ribose) polymerase inhibitor
PARylation	poly(ADP-ribosyl)ation
PDEF	prostate-derived Ets factor
PD-L1	programmed cell death-ligant 1
PEA3	polyomavirus enhancer activator 3 homolog
RNA	ribonucleic acid
RNF146	RING finger protein 146
ROS	reactive oxygen species
Spdef	SAM pointed domain-containing Ets transcription factor
SSB	single-strand break
TCF	ternary complex factor
TMPRSS2	transmembrane protease serine 2
WT	Wild type

## References

[1] Degnan B.M., Degnan S.M., Naganuma T., Morse D.E. (1993). The *ets* Multigene Family Is Conserved throughout the Metazoa. Nucl. Acids Res..

[2] Laudet V., Hänni C., Stéhelin D., Duterque-Coquillaud M. (1999). Molecular Phylogeny of the ETS Gene Family. Oncogene.

[3] Hollenhorst P.C., McIntosh L.P., Graves B.J. (2011). Genomic and Biochemical Insights into the Specificity of ETS Transcription Factors. Annu. Rev. Biochem..

[4] Wei G.-H., Badis G., Berger M.F., Kivioja T., Palin K., Enge M., Bonke M., Jolma A., Varjosalo M., Gehrke A.R. (2010). Genome-Wide Analysis of ETS-Family DNA-Binding in Vitro and in Vivo. EMBO J..

[5] Oikawa T., Yamada T. (2003). Molecular Biology of the Ets Family of Transcription Factors. Gene.

[6] Seth A., Watson D.K. (2005). ETS Transcription Factors and Their Emerging Roles in Human Cancer. Eur. J. Cancer.

[7] Hsu T., Trojanowska M., Watson D.K. (2004). Ets Proteins in Biological Control and Cancer. J. Cell Biochem..

[8] Shao L., Tekedereli I., Wang J., Yuca E., Tsang S., Sood A., Lopez-Berestein G., Ozpolat B., Ittmann M. (2012). Highly Specific Targeting of the TMPRSS2/ERG Fusion Gene Using Liposomal Nanovectors. Clin. Cancer Res..

[9] Li L., Hobson L., Perry L., Clark B., Heavey S., Haider A., Sridhar A., Shaw G., Kelly J., Freeman A. (2020). Targeting the ERG Oncogene with Splice-Switching Oligonucleotides as a Novel Therapeutic Strategy in Prostate Cancer. Br. J. Cancer.

[10] Laitem C., Leprivier G., Choul-Li S., Begue A., Monte D., Larsimont D., Dumont P., Duterque-Coquillaud M., Aumercier M. (2009). Ets-1 P27: A Novel Ets-1 Isoform with Dominant-Negative Effects on the Transcriptional Properties and the Subcellular Localization of Ets-1 P51. Oncogene.

[11] Sahin A., Vercamer C., Kaminski A., Fuchs T., Florin A., Hahne J.C., Mattot V., Pourtier-Manzanedo A., Pietsch T., Fafeur V. (2009). Dominant-Negative Inhibition of Ets 1 Suppresses Tumor Growth, Invasion and Migration in Rat C6 Glioma Cells and Reveals Differentially Expressed Ets 1 Target Genes. Int. J. Oncol..

[12] Wang X., Qiao Y., Asangani I.A., Ateeq B., Poliakov A., Cieślik M., Pitchiaya S., Chakravarthi B.V.S.K., Cao X., Jing X. (2017). Development of Peptidomimetic Inhibitors of the ERG Gene Fusion Product in Prostate Cancer. Cancer Cell.

[13] Nhili R., Peixoto P., Depauw S., Flajollet S., Dezitter X., Munde M.M., Ismail M.A., Kumar A., Farahat A.A., Stephens C.E. (2013). Targeting the DNA-Binding Activity of the Human ERG Transcription Factor Using New Heterocyclic Dithiophene Diamidines. Nucleic Acids Res..

[14] Harlow M.L., Chasse M.H., Boguslawski E.A., Sorensen K.M., Gedminas J.M., Kitchen-Goosen S.M., Rothbart S.B., Taslim C., Lessnick S.L., Peck A.S. (2019). Trabectedin Inhibits EWS-FLI1 and Evicts SWI/SNF from Chromatin in a Schedule-Dependent Manner. Clin. Cancer Res..

[15] Harlow M.L., Maloney N., Roland J., Guillen Navarro M.J., Easton M.K., Kitchen-Goosen S.M., Boguslawski E.A., Madaj Z.B., Johnson B.K., Bowman M.J. (2016). Lurbinectedin Inactivates the Ewing Sarcoma Oncoprotein EWS-FLI1 by Redistributing It within the Nucleus. Cancer Res..

[16] Erkizan H.V., Kong Y., Merchant M., Schlottmann S., Barber-Rotenberg J.S., Yuan L., Abaan O.D., Chou T.-H., Dakshanamurthy S., Brown M.L. (2009). A Small Molecule Blocking Oncogenic Protein EWS-FLI1 Interaction with RNA Helicase A Inhibits Growth of Ewing’s Sarcoma. Nat. Med..

[17] Rosati R., Polin L., Ducker C., Li J., Bao X., Selvakumar D., Kim S., Xhabija B., Larsen M., McFall T. (2018). Strategy for Tumor-Selective Disruption of Androgen Receptor Function in the Spectrum of Prostate Cancer. Clin. Cancer Res..

[18] Butler M.S., Roshan-Moniri M., Hsing M., Lau D., Kim A., Yen P., Mroczek M., Nouri M., Lien S., Axerio-Cilies P. (2017). Discovery and Characterization of Small Molecules Targeting the DNA-Binding ETS Domain of ERG in Prostate Cancer. Oncotarget.

[19] Rahim S., Beauchamp E.M., Kong Y., Brown M.L., Toretsky J.A., Üren A. (2011). YK-4-279 Inhibits ERG and ETV1 Mediated Prostate Cancer Cell Invasion. PLoS ONE.

[20] Rahim S., Minas T., Hong S.-H., Justvig S., Çelik H., Kont Y.S., Han J., Kallarakal A.T., Kong Y., Rudek M.A. (2014). A Small Molecule Inhibitor of ETV1, YK-4-279, Prevents Prostate Cancer Growth and Metastasis in a Mouse Xenograft Model. PLoS ONE.

[21] Pop M.S., Stransky N., Garvie C.W., Theurillat J.-P., Hartman E.C., Lewis T.A., Zhong C., Culyba E.K., Lin F., Daniels D.S. (2014). A Small Molecule That Binds and Inhibits the ETV1 Transcription Factor Oncoprotein. Mol. Cancer Ther..

[22] Liu T., Xia L., Yao Y., Yan C., Fan Y., Gajendran B., Yang J., Li Y.-J., Chen J., Filmus J. (2019). Identification of Diterpenoid Compounds That Interfere with Fli-1 DNA Binding to Suppress Leukemogenesis. Cell Death Dis..

[23] Liu Y., Eckenrode J.M., Zhang Y., Zhang J., Hayden R.C., Kyomuhangi A., Ponomareva L.V., Cui Z., Rohr J., Tsodikov O.V. (2020). Mithramycin 2′-Oximes with Improved Selectivity, Pharmacokinetics, and Ewing Sarcoma Antitumor Efficacy. J. Med. Chem..

[24] Mitra P., Eckenrode J.M., Mandal A., Jha A.K., Salem S.M., Leggas M., Rohr J. (2018). Development of Mithramycin Analogues with Increased Selectivity toward ETS Transcription Factor Expressing Cancers. J. Med. Chem..

[25] Mohamed A.A., Xavier C.P., Sukumar G., Tan S.-H., Ravindranath L., Seraj N., Kumar V., Sreenath T., McLeod D.G., Petrovics G. (2018). Identification of a Small Molecule That Selectively Inhibits ERG-Positive Cancer Cell Growth. Cancer Res..

[26] Grohar P.J., Glod J., Peer C.J., Sissung T.M., Arnaldez F.I., Long L., Figg W.D., Whitcomb P., Helman L.J., Widemann B.C. (2017). A Phase I/II Trial and Pharmacokinetic Study of Mithramycin in Children and Adults with Refractory Ewing Sarcoma and EWS-FLI1 Fusion Transcript. Cancer Chemother. Pharmacol..

[27] Brenner J.C., Feng F.Y., Han S., Patel S., Goyal S.V., Bou-Maroun L.M., Liu M., Lonigro R., Prensner J.R., Tomlins S.A. (2012). PARP-1 Inhibition as a Targeted Strategy to Treat Ewing’s Sarcoma. Cancer Res..

[28] Brenner J.C., Ateeq B., Li Y., Yocum A.K., Cao Q., Asangani I.A., Patel S., Wang X., Liang H., Yu J. (2011). Mechanistic Rationale for Inhibition of Poly(ADP-Ribose) Polymerase in ETS Gene Fusion-Positive Prostate Cancer. Cancer Cell.

[29] Legrand A.J., Choul-Li S., Spriet C., Idziorek T., Vicogne D., Drobecq H., Dantzer F., Villeret V., Aumercier M. (2013). The Level of Ets-1 Protein Is Regulated by Poly(ADP-Ribose) Polymerase-1 (PARP-1) in Cancer Cells to Prevent DNA Damage. PLoS ONE.

[30] Gelmon K.A., Tischkowitz M., Mackay H., Swenerton K., Robidoux A., Tonkin K., Hirte H., Huntsman D., Clemons M., Gilks B. (2011). Olaparib in Patients with Recurrent High-Grade Serous or Poorly Differentiated Ovarian Carcinoma or Triple-Negative Breast Cancer: A Phase 2, Multicentre, Open-Label, Non-Randomised Study. Lancet Oncol..

[31] Loap P., Loirat D., Berger F., Cao K., Ricci F., Jochem A., Raizonville L., Mosseri V., Fourquet A., Kirova Y. (2021). Combination of Olaparib with Radiotherapy for Triple-negative Breast Cancers: One-year Toxicity Report of the RADIOPARP Phase I Trial. Int. J. Cancer.

[32] Wu Y., Xu S., Cheng S., Yang J., Wang Y. (2023). Clinical Application of PARP Inhibitors in Ovarian Cancer: From Molecular Mechanisms to the Current Status. J. Ovarian Res..

[33] Leprince D., Gegonne A., Coll J., de Taisne C., Schneeberger A., Lagrou C., Stehelin D. (1983). A Putative Second Cell-Derived Oncogene of the Avian Leukaemia Retrovirus E26. Nature.

[34] Nunn M.F., Seeburg P.H., Moscovici C., Duesberg P.H. (1983). Tripartite Structure of the Avian Erythroblastosis Virus E26 Transforming Gene. Nature.

[35] Radke K., Beug H., Kornfeld S., Graf T. (1982). Transformation of Both Erythroid and Myeloid Cells by E26, an Avian Leukemia Virus That Contains the Myb Gene. Cell.

[36] Hsing M., Wang Y., Rennie P.S., Cox M.E., Cherkasov A. (2020). ETS Transcription Factors as Emerging Drug Targets in Cancer. Med. Res. Rev..

[37] Powell K., Semaan L., Conley-LaComb M.K., Asangani I., Wu Y.-M., Ginsburg K.B., Williams J., Squire J.A., Maddipati K.R., Cher M.L. (2015). ERG/AKR1C3/AR Constitutes a Feed-Forward Loop for AR Signaling in Prostate Cancer Cells. Clin. Cancer Res..

[38] Kollareddy M., Dimitrova E., Vallabhaneni K.C., Chan A., Le T., Chauhan K.M., Carrero Z.I., Ramakrishnan G., Watabe K., Haupt Y. (2015). Regulation of Nucleotide Metabolism by Mutant P53 Contributes to Its Gain-of-Function Activities. Nat. Commun..

[39] Kar A., Gutierrez-Hartmann A. (2013). Molecular Mechanisms of ETS Transcription Factor-Mediated Tumorigenesis. Crit. Rev. Biochem. Mol. Biol..

[40] Zhu J., Sammons M.A., Donahue G., Dou Z., Vedadi M., Getlik M., Barsyte-Lovejoy D., Al-awar R., Katona B.W., Shilatifard A. (2015). Gain-of-Function P53 Mutants Co-Opt Chromatin Pathways to Drive Cancer Growth. Nature.

[41] Chakrabarti R., Hwang J., Andres Blanco M., Wei Y., Lukačišin M., Romano R.-A., Smalley K., Liu S., Yang Q., Ibrahim T. (2012). Elf5 Inhibits the Epithelial–Mesenchymal Transition in Mammary Gland Development and Breast Cancer Metastasis by Transcriptionally Repressing Snail2. Nat. Cell Biol..

[42] Chatterjee P., Choudhary G.S., Alswillah T., Xiong X., Heston W.D., Magi-Galluzzi C., Zhang J., Klein E.A., Almasan A. (2015). The TMPRSS2–ERG Gene Fusion Blocks XRCC4-Mediated Nonhomologous End-Joining Repair and Radiosensitizes Prostate Cancer Cells to PARP Inhibition. Mol. Cancer Ther..

[43] Buchwalter G., Hickey M.M., Cromer A., Selfors L.M., Gunawardane R.N., Frishman J., Jeselsohn R., Lim E., Chi D., Fu X. (2013). PDEF Promotes Luminal Differentiation and Acts as a Survival Factor for ER-Positive Breast Cancer Cells. Cancer Cell.

[44] Noah T.K., Lo Y., Price A., Chen G., King E., Washington M., Aronow B.J., Shroyer N.F. (2013). SPDEF Functions as a Colorectal Tumor Suppressor by Inhibiting β-Catenin Activity. Gastroenterology.

[45] Steffan J.J., Koul S., Meacham R.B., Koul H.K. (2016). The Transcription Factor SPDEF Suppresses Prostate Tumor Metastasis. J. Biol. Chem..

[46] Cohen-Armon M., Visochek L., Rozensal D., Kalal A., Geistrikh I., Klein R., Bendetz-Nezer S., Yao Z., Seger R. (2007). DNA-Independent PARP-1 Activation by Phosphorylated ERK2 Increases Elk1 Activity: A Link to Histone Acetylation. Mol. Cell.

[47] Wang H., Fang R., Cho J.-Y., Libermann T.A., Oettgen P. (2004). Positive and Negative Modulation of the Transcriptional Activity of the ETS Factor ESE-1 through Interaction with P300, CREB-Binding Protein, and Ku 70/86. J. Biol. Chem..

[48] Zaniolo K., Desnoyers S., Leclerc S., Guérin S.L. (2007). Regulation of Poly(ADP-Ribose) Polymerase-1 (PARP-1) Gene Expression through the Post-Translational Modification of Sp1: A Nuclear Target Protein of PARP-1. BMC Mol. Biol..

[49] Lessnick S.L., Ladanyi M. (2012). Molecular Pathogenesis of Ewing Sarcoma: New Therapeutic and Transcriptional Targets. Annu. Rev. Pathol. Mech. Dis..

[50] Tomlins S.A., Bjartell A., Chinnaiyan A.M., Jenster G., Nam R.K., Rubin M.A., Schalken J.A. (2009). ETS Gene Fusions in Prostate Cancer: From Discovery to Daily Clinical Practice. Eur. Urol..

[51] Qian C., Li D., Chen Y. (2022). ETS Factors in Prostate Cancer. Cancer Lett..

[52] De Braekeleer E., Douet-Guilbert N., Morel F., Le Bris M.-J., Basinko A., De Braekeleer M. (2012). ETV6 Fusion Genes in Hematological Malignancies: A Review. Leuk. Res..

[53] Apfelbaum A.A., Wrenn E.D., Lawlor E.R. (2022). The Importance of Fusion Protein Activity in Ewing Sarcoma and the Cell Intrinsic and Extrinsic Factors That Regulate It: A Review. Front. Oncol..

[54] Tomlins S.A., Rhodes D.R., Perner S., Dhanasekaran S.M., Mehra R., Sun X.-W., Varambally S., Cao X., Tchinda J., Kuefer R. (2005). Recurrent Fusion of *TMPRSS2* and ETS Transcription Factor Genes in Prostate Cancer. Science.

[55] Khosh Kish E., Choudhry M., Gamallat Y., Buharideen S.M., Bismar T.A. (2022). The Expression of Proto-Oncogene ETS-Related Gene (ERG) Plays a Central Role in the Oncogenic Mechanism Involved in the Development and Progression of Prostate Cancer. Int. J. Mol. Sci..

[56] Nicholas T.R., Strittmatter B.G., Hollenhorst P.C., Dehm S.M., Tindall D.J. (2019). Oncogenic ETS Factors in Prostate Cancer. Prostate Cancer.

[57] Kim M.Y., Zhang T., Kraus W.L. (2005). Poly(ADP-Ribosyl)Ation by PARP-1: ‘PAR-Laying’ NAD ^+^ into a Nuclear Signal. Genes Dev..

[58] De Vos M., Schreiber V., Dantzer F. (2012). The Diverse Roles and Clinical Relevance of PARPs in DNA Damage Repair: Current State of the Art. Biochem. Pharmacol..

[59] Ray Chaudhuri A., Nussenzweig A. (2017). The Multifaceted Roles of PARP1 in DNA Repair and Chromatin Remodelling. Nat. Rev. Mol. Cell Biol..

[60] Luo X., Kraus W.L. (2012). On PAR with PARP: Cellular Stress Signaling through Poly(ADP-Ribose) and PARP-1. Genes Dev..

[61] Kraus W.L. (2008). Transcriptional Control by PARP-1: Chromatin Modulation, Enhancer-Binding, Coregulation, and Insulation. Curr. Opin. Cell Biol..

[62] Ogino H., Nozaki T., Gunji A., Maeda M., Suzuki H., Ohta T., Murakami Y., Nakagama H., Sugimura T., Masutani M. (2007). Loss of Parp-1 Affects Gene Expression Profile in a Genome-Wide Manner in ES Cells and Liver Cells. BMC Genom..

[63] Masutani M., Nakagama H., Sugimura T. (2005). Poly(ADP-Ribosyl)Ation in Relation to Cancer and Autoimmune Disease. Cell Mol. Life Sci..

[64] Akanksha, Mishra S., Kar A., Karthik J., Srivastava A., Khanna R., Meena R. (2022). Expression of Poly(Adenosine Diphosphate-Ribose) Polymerase Protein in Breast Cancer. J. Mid-Life Health.

[65] Bièche I., de Murcia G., Lidereau R. (1996). Poly(ADP-Ribose) Polymerase Gene Expression Status and Genomic Instability in Human Breast Cancer. Clin. Cancer Res..

[66] Quiles-Perez R., Muñoz-Gámez J.A., Ruiz-Extremera Á., O’Valle F., Sanjuán-Nuñez L., Martín-Álvarez A.B., Martín-Oliva D., Caballero T., Muñoz de Rueda P., León J. (2010). Inhibition of Poly Adenosine Diphosphate-Ribose Polymerase Decreases Hepatocellular Carcinoma Growth by Modulation of Tumor-Related Gene Expression. Hepatology.

[67] Nosho K., Yamamoto H., Mikami M., Taniguchi H., Takahashi T., Adachi Y., Imamura A., Imai K., Shinomura Y. (2006). Overexpression of Poly(ADP-Ribose) Polymerase-1 (PARP-1) in the Early Stage of Colorectal Carcinogenesis. Eur. J. Cancer.

[68] Staibano S., Pepe S., Muzio L.L., Somma P., Mascolo M., Argenziano G., Scalvenzi M., Salvatore G., Fabbrocini G., Molea G. (2005). Poly(Adenosine Diphosphate-Ribose) Polymerase 1 Expression in Malignant Melanomas from Photoexposed Areas of the Head and Neck Region. Hum. Pathol..

[69] Masutani M., Fujimori H. (2013). Poly(ADP-Ribosyl)Ation in Carcinogenesis. Mol. Asp. Med..

[70] Zaremba T., Ketzer P., Cole M., Coulthard S., Plummer E.R., Curtin N.J. (2009). Poly(ADP-Ribose) Polymerase-1 Polymorphisms, Expression and Activity in Selected Human Tumour Cell Lines. Br. J. Cancer.

[71] Dréan A., Lord C.J., Ashworth A. (2016). PARP Inhibitor Combination Therapy. Crit. Rev. Oncol. Hematol..

[72] Curtin N.J., Szabo C. (2020). Poly(ADP-Ribose) Polymerase Inhibition: Past, Present and Future. Nat. Rev. Drug Discov..

[73] Farmer H., McCabe N., Lord C.J., Tutt A.N.J., Johnson D.A., Richardson T.B., Santarosa M., Dillon K.J., Hickson I., Knights C. (2005). Targeting the DNA Repair Defect in BRCA Mutant Cells as a Therapeutic Strategy. Nature.

[74] Bryant H.E., Schultz N., Thomas H.D., Parker K.M., Flower D., Lopez E., Kyle S., Meuth M., Curtin N.J., Helleday T. (2005). Specific Killing of BRCA2-Deficient Tumours with Inhibitors of Poly(ADP-Ribose) Polymerase. Nature.

[75] Yap T.A., Sandhu S.K., Carden C.P., de Bono J.S. (2011). Poly(ADP-Ribose) Polymerase (PARP) Inhibitors: Exploiting a Synthetic Lethal Strategy in the Clinic. CA Cancer J. Clin..

[76] Fong P.C., Boss D.S., Yap T.A., Tutt A., Wu P., Mergui-Roelvink M., Mortimer P., Swaisland H., Lau A., O’Connor M.J. (2009). Inhibition of poly (ADP-ribose) polymerase in tumors from BRCA mutation carriers. N. Engl. J. Med..

[77] McCabe N., Turner N.C., Lord C.J., Kluzek K., Białkowska A., Swift S., Giavara S., O’Connor M.J., Tutt A.N., Zdzienicka M.Z. (2006). Deficiency in the Repair of DNA Damage by Homologous Recombination and Sensitivity to Poly(ADP-Ribose) Polymerase Inhibition. Cancer Res..

[78] Ledermann J., Harter P., Gourley C., Friedlander M., Vergote I., Rustin G., Scott C.L., Meier W., Shapira-Frommer R., Safra T. (2014). Olaparib Maintenance Therapy in Patients with Platinum-Sensitive Relapsed Serous Ovarian Cancer: A Preplanned Retrospective Analysis of Outcomes by BRCA Status in a Randomised Phase 2 Trial. Lancet Oncol..

[79] Pujade-Lauraine E., Ledermann J.A., Selle F., Gebski V., Penson R.T., Oza A.M., Korach J., Huzarski T., Poveda A., Pignata S. (2017). Olaparib Tablets as Maintenance Therapy in Patients with Platinum-Sensitive, Relapsed Ovarian Cancer and a BRCA1/2 Mutation (SOLO2/ENGOT-Ov21): A Double-Blind, Randomised, Placebo-Controlled, Phase 3 Trial. Lancet Oncol..

[80] Moore K., Colombo N., Scambia G., Kim B.-G., Oaknin A., Friedlander M., Lisyanskaya A., Floquet A., Leary A., Sonke G.S. (2018). Maintenance Olaparib in Patients with Newly Diagnosed Advanced Ovarian Cancer. N. Engl. J. Med..

[81] Robson M., Im S.-A., Senkus E., Xu B., Domchek S.M., Masuda N., Delaloge S., Li W., Tung N., Armstrong A. (2017). Olaparib for Metastatic Breast Cancer in Patients with a Germline BRCA Mutation. N. Engl. J. Med..

[82] Golan T., Hammel P., Reni M., Van Cutsem E., Macarulla T., Hall M.J., Park J.-O., Hochhauser D., Arnold D., Oh D.-Y. (2019). Maintenance Olaparib for Germline BRCA-Mutated Metastatic Pancreatic Cancer. N. Engl. J. Med..

[83] Plummer R., Jones C., Middleton M., Wilson R., Evans J., Olsen A., Curtin N., Boddy A., McHugh P., Newell D. (2008). Phase I Study of the Poly(ADP-Ribose) Polymerase Inhibitor, AG014699, in Combination with Temozolomide in Patients with Advanced Solid Tumors. Clin. Cancer Res..

[84] Kristeleit R., Shapiro G.I., Burris H.A., Oza A.M., LoRusso P., Patel M.R., Domchek S.M., Balmaña J., Drew Y., Chen L.-M. (2017). A Phase I-II Study of the Oral PARP Inhibitor Rucaparib in Patients with Germline BRCA1/2-Mutated Ovarian Carcinoma or Other Solid Tumors. Clin. Cancer Res..

[85] Swisher E.M., Lin K.K., Oza A.M., Scott C.L., Giordano H., Sun J., Konecny G.E., Coleman R.L., Tinker A.V., O’Malley D.M. (2017). Rucaparib in Relapsed, Platinum-Sensitive High-Grade Ovarian Carcinoma (ARIEL2 Part 1): An International, Multicentre, Open-Label, Phase 2 Trial. Lancet Oncol..

[86] Coleman R.L., Oza A.M., Lorusso D., Aghajanian C., Oaknin A., Dean A., Colombo N., Weberpals J.I., Clamp A., Scambia G. (2017). Rucaparib Maintenance Treatment for Recurrent Ovarian Carcinoma after Response to Platinum Therapy (ARIEL3): A Randomised, Double-Blind, Placebo-Controlled, Phase 3 Trial. Lancet.

[87] Oza A.M., Tinker A.V., Oaknin A., Shapira-Frommer R., McNeish I.A., Swisher E.M., Ray-Coquard I., Bell-McGuinn K., Coleman R.L., O’Malley D.M. (2017). Antitumor Activity and Safety of the PARP Inhibitor Rucaparib in Patients with High-Grade Ovarian Carcinoma and a Germline or Somatic BRCA1 or BRCA2 Mutation: Integrated Analysis of Data from Study 10 and ARIEL2. Gynecol. Oncol..

[88] Moore K.N., Secord A.A., Geller M.A., Miller D.S., Cloven N., Fleming G.F., Wahner Hendrickson A.E., Azodi M., DiSilvestro P., Oza A.M. (2019). Niraparib Monotherapy for Late-Line Treatment of Ovarian Cancer (QUADRA): A Multicentre, Open-Label, Single-Arm, Phase 2 Trial. Lancet Oncol..

[89] Mirza M.R., Monk B.J., Herrstedt J., Oza A.M., Mahner S., Redondo A., Fabbro M., Ledermann J.A., Lorusso D., Vergote I. (2016). Niraparib Maintenance Therapy in Platinum-Sensitive, Recurrent Ovarian Cancer. N. Engl. J. Med..

[90] Litton J.K., Rugo H.S., Ettl J., Hurvitz S.A., Gonçalves A., Lee K.-H., Fehrenbacher L., Yerushalmi R., Mina L.A., Martin M. (2018). Talazoparib in Patients with Advanced Breast Cancer and a Germline *BRCA* Mutation. N. Engl. J. Med..

[91] Dias M.P., Moser S.C., Ganesan S., Jonkers J. (2021). Understanding and Overcoming Resistance to PARP Inhibitors in Cancer Therapy. Nat. Rev. Clin. Oncol..

[92] Noordermeer S.M., van Attikum H. (2019). PARP Inhibitor Resistance: A Tug-of-War in BRCA-Mutated Cells. Trends Cell Biol..

[93] Rottenberg S., Jaspers J.E., Kersbergen A., van der Burg E., Nygren A.O.H., Zander S.A.L., Derksen P.W.B., de Bruin M., Zevenhoven J., Lau A. (2008). High Sensitivity of BRCA1-Deficient Mammary Tumors to the PARP Inhibitor AZD2281 Alone and in Combination with Platinum Drugs. Proc. Natl. Acad. Sci. USA.

[94] Jaspers J.E., Kersbergen A., Boon U., Sol W., Van Deemter L., Zander S.A., Drost R., Wientjens E., Ji J., Aly A. (2013). Loss of 53BP1 Causes PARP Inhibitor Resistance in *Brca1*-Mutated Mouse Mammary Tumors. Cancer Discov..

[95] Gogola E., Duarte A.A., de Ruiter J.R., Wiegant W.W., Schmid J.A., de Bruijn R., James D.I., Guerrero Llobet S., Vis D.J., Annunziato S. (2018). Selective Loss of PARG Restores PARylation and Counteracts PARP Inhibitor-Mediated Synthetic Lethality. Cancer Cell.

[96] Pettitt S.J., Krastev D.B., Brandsma I., Dréan A., Song F., Aleksandrov R., Harrell M.I., Menon M., Brough R., Campbell J. (2018). Genome-Wide and High-Density CRISPR-Cas9 Screens Identify Point Mutations in PARP1 Causing PARP Inhibitor Resistance. Nat. Commun..

[97] Johnson N., Johnson S.F., Yao W., Li Y.-C., Choi Y.-E., Bernhardy A.J., Wang Y., Capelletti M., Sarosiek K.A., Moreau L.A. (2013). Stabilization of Mutant BRCA1 Protein Confers PARP Inhibitor and Platinum Resistance. Proc. Natl. Acad. Sci. USA.

[98] Ter Brugge P., Kristel P., Van Der Burg E., Boon U., De Maaker M., Lips E., Mulder L., De Ruiter J., Moutinho C., Gevensleben H. (2016). Mechanisms of Therapy Resistance in Patient-Derived Xenograft Models of BRCA1-Deficient Breast Cancer. JNCI J. Natl. Cancer Inst..

[99] Kondrashova O., Topp M., Nesic K., Lieschke E., Ho G.-Y., Harrell M.I., Zapparoli G.V., Hadley A., Holian R., Boehm E. (2018). Methylation of All BRCA1 Copies Predicts Response to the PARP Inhibitor Rucaparib in Ovarian Carcinoma. Nat. Commun..

[100] Nacson J., Krais J.J., Bernhardy A.J., Clausen E., Feng W., Wang Y., Nicolas E., Cai K.Q., Tricarico R., Hua X. (2018). BRCA1 Mutation-Specific Responses to 53BP1 Loss-Induced Homologous Recombination and PARP Inhibitor Resistance. Cell Rep..

[101] Dev H., Chiang T.-W.W., Lescale C., de Krijger I., Martin A.G., Pilger D., Coates J., Sczaniecka-Clift M., Wei W., Ostermaier M. (2018). Shieldin Complex Promotes DNA End-Joining and Counters Homologous Recombination in BRCA1-Null Cells. Nat. Cell Biol..

[102] Tomida J., Takata K., Bhetawal S., Person M.D., Chao H., Tang D.G., Wood R.D. (2018). FAM 35A Associates with REV 7 and Modulates DNA Damage Responses of Normal and BRCA 1-defective Cells. EMBO J..

[103] Xu G., Chapman J.R., Brandsma I., Yuan J., Mistrik M., Bouwman P., Bartkova J., Gogola E., Warmerdam D., Barazas M. (2015). REV7 Counteracts DNA Double-Strand Break Resection and Affects PARP Inhibition. Nature.

[104] Dungrawala H., Bhat K.P., Le Meur R., Chazin W.J., Ding X., Sharan S.K., Wessel S.R., Sathe A.A., Zhao R., Cortez D. (2017). RADX Promotes Genome Stability and Modulates Chemosensitivity by Regulating RAD51 at Replication Forks. Mol. Cell.

[105] Rondinelli B., Gogola E., Yücel H., Duarte A.A., Van De Ven M., Van Der Sluijs R., Konstantinopoulos P.A., Jonkers J., Ceccaldi R., Rottenberg S. (2017). EZH2 Promotes Degradation of Stalled Replication Forks by Recruiting MUS81 through Histone H3 Trimethylation. Nat. Cell Biol..

[106] Ray Chaudhuri A., Callen E., Ding X., Gogola E., Duarte A.A., Lee J.-E., Wong N., Lafarga V., Calvo J.A., Panzarino N.J. (2016). Replication Fork Stability Confers Chemoresistance in BRCA-Deficient Cells. Nature.

[107] Murai J., Feng Y., Yu G.K., Ru Y., Tang S.-W., Shen Y., Pommier Y. (2016). Resistance to PARP Inhibitors by SLFN11 Inactivation Can Be Overcome by ATR Inhibition. Oncotarget.

[108] Kim H., Xu H., George E., Hallberg D., Kumar S., Jagannathan V., Medvedev S., Kinose Y., Devins K., Verma P. (2020). Combining PARP with ATR Inhibition Overcomes PARP Inhibitor and Platinum Resistance in Ovarian Cancer Models. Nat. Commun..

[109] Westin S.N., Coleman R.L., Fellman B.M., Yuan Y., Sood A.K., Soliman P.T., Wright A.A., Horowitz N.S., Campos S.M., Konstantinopoulos P.A. (2021). EFFORT: EFFicacy Of Adavosertib in Parp ResisTance: A Randomized Two-Arm Non-Comparative Phase II Study of Adavosertib with or without Olaparib in Women with PARP-Resistant Ovarian Cancer. J. Clin. Oncol..

[110] Fang Y., McGrail D.J., Sun C., Labrie M., Chen X., Zhang D., Ju Z., Vellano C.P., Lu Y., Li Y. (2019). Sequential Therapy with PARP and WEE1 Inhibitors Minimizes Toxicity While Maintaining Efficacy. Cancer Cell.

[111] Jiao S., Xia W., Yamaguchi H., Wei Y., Chen M.-K., Hsu J.-M., Hsu J.L., Yu W.-H., Du Y., Lee H.-H. (2017). PARP Inhibitor Upregulates PD-L1 Expression and Enhances Cancer-Associated Immunosuppression. Clin. Cancer Res..

[112] Du Y., Yamaguchi H., Wei Y., Hsu J.L., Wang H.-L., Hsu Y.-H., Lin W.-C., Yu W.-H., Leonard P.G., Lee G.R. (2016). Blocking C-Met–Mediated PARP1 Phosphorylation Enhances Anti-Tumor Effects of PARP Inhibitors. Nat. Med..

[113] Soldatenkov V.A., Albor A., Patel B.K., Dreszer R., Dritschilo A., Notario V. (1999). Regulation of the Human Poly(ADP-Ribose) Polymerase Promoter by the ETS Transcription Factor. Oncogene.

[114] Soldatenkov V.A., Trofimova I.N., Rouzaut A., McDermott F., Dritschilo A., Notario V. (2002). Differential Regulation of the Response to DNA Damage in Ewing’s Sarcoma Cells by ETS1 and EWS/FLI-1. Oncogene.

[115] Li D., Bi F.-F., Cao J.-M., Cao C., Li C.-Y., Liu B., Yang Q. (2014). Poly (ADP-Ribose) Polymerase 1 Transcriptional Regulation: A Novel Crosstalk between Histone Modification H3K9ac and ETS1 Motif Hypomethylation in BRCA1-Mutated Ovarian Cancer. Oncotarget.

[116] Molloy-Simard V., St-Laurent J.-F., Vigneault F., Gaudreault M., Dargis N., Guérin M.-C., Leclerc S., Morcos M., Black D., Molgat Y. (2012). Altered Expression of the Poly(ADP-Ribosyl)Ation Enzymes in Uveal Melanoma and Regulation of *PARG* Gene Expression by the Transcription Factor ERM. Investig. Ophthalmol. Vis. Sci..

[117] Choul-li S., Legrand A.J., Bidon B., Vicogne D., Villeret V., Aumercier M. (2018). Ets-1 Interacts through a Similar Binding Interface with Ku70 and Poly (ADP-Ribose) Polymerase-1. Biosci. Biotechnol. Biochem..

[118] Ibrahim Y.H., García-García C., Serra V., He L., Torres-Lockhart K., Prat A., Anton P., Cozar P., Guzmán M., Grueso J. (2012). PI3K Inhibition Impairs BRCA1/2 Expression and Sensitizes BRCA-Proficient Triple-Negative Breast Cancer to PARP Inhibition. Cancer Discov..

[119] Han S., Brenner J.C., Sabolch A., Jackson W., Speers C., Wilder-Romans K., Knudsen K.E., Lawrence T.S., Chinnaiyan A.M., Feng F.Y. (2013). Targeted Radiosensitization of ETS Fusion-Positive Prostate Cancer through PARP1 Inhibition. Neoplasia.

[120] Kalisch T., Amé J.-C., Dantzer F., Schreiber V. (2012). New Readers and Interpretations of Poly(ADP-Ribosyl)Ation. Trends Biochem. Sci..

[121] Chatterjee P., Choudhary G.S., Sharma A., Singh K., Heston W.D., Ciezki J., Klein E.A., Almasan A. (2013). PARP Inhibition Sensitizes to Low Dose-Rate Radiation TMPRSS2-ERG Fusion Gene-Expressing and PTEN-Deficient Prostate Cancer Cells. PLoS ONE.

[122] Garnett M.J., Edelman E.J., Heidorn S.J., Greenman C.D., Dastur A., Lau K.W., Greninger P., Thompson I.R., Luo X., Soares J. (2012). Systematic Identification of Genomic Markers of Drug Sensitivity in Cancer Cells. Nature.

[123] Lovejoy C.A., Xu X., Bansbach C.E., Glick G.G., Zhao R., Ye F., Sirbu B.M., Titus L.C., Shyr Y., Cortez D. (2009). Functional Genomic Screens Identify CINP as a Genome Maintenance Protein. Proc. Natl. Acad. Sci. USA..

[124] Ni W., Zhan Y., He H., Maynard E., Balschi J.A., Oettgen P. (2007). Ets-1 Is a Critical Transcriptional Regulator of Reactive Oxygen Species and P47(Phox) Gene Expression in Response to Angiotensin II. Circ. Res..

[125] Baillat D., Bègue A., Stéhelin D., Aumercier M. (2002). ETS-1 Transcription Factor Binds Cooperatively to the Palindromic Head to Head ETS-Binding Sites of the Stromelysin-1 Promoter by Counteracting Autoinhibition. J. Biol. Chem..

[126] Baillat D., Leprivier G., Régnier D., Vintonenko N., Bègue A., Stéhelin D., Aumercier M. (2006). Stromelysin-1 Expression Is Activated in Vivo by Ets-1 through Palindromic Head-to-Head Ets Binding Sites Present in the Promoter. Oncogene.

[127] Radisky D.C., Levy D.D., Littlepage L.E., Liu H., Nelson C.M., Fata J.E., Leake D., Godden E.L., Albertson D.G., Angela Nieto M. (2005). Rac1b and Reactive Oxygen Species Mediate MMP-3-Induced EMT and Genomic Instability. Nature.

[128] Singh A.K., Swarnalatha M., Kumar V. (2011). C-ETS1 Facilitates G1/S-Phase Transition by Up-Regulating Cyclin E and CDK2 Genes and Cooperates with Hepatitis B Virus X Protein for Their Deregulation. J. Biol. Chem..

[129] Ju B.-G., Lunyak V.V., Perissi V., Garcia-Bassets I., Rose D.W., Glass C.K., Rosenfeld M.G. (2006). A Topoisomerase IIß-Mediated DsDNA Break Required for Regulated Transcription. Science.

[130] Dominguez-Sola D., Ying C.Y., Grandori C., Ruggiero L., Chen B., Li M., Galloway D.A., Gu W., Gautier J., Dalla-Favera R. (2007). Non-Transcriptional Control of DNA Replication by c-Myc. Nature.

[131] Sandhu S.K., Schelman W.R., Wilding G., Moreno V., Baird R.D., Miranda S., Hylands L., Riisnaes R., Forster M., Omlin A. (2013). The Poly(ADP-Ribose) Polymerase Inhibitor Niraparib (MK4827) in BRCA Mutation Carriers and Patients with Sporadic Cancer: A Phase 1 Dose-Escalation Trial. Lancet Oncol..

[132] Lee H.-J., Yoon C., Schmidt B., Park D.J., Zhang A.Y., Erkizan H.V., Toretsky J.A., Kirsch D.G., Yoon S.S. (2013). Combining PARP-1 Inhibition and Radiation in Ewing Sarcoma Results in Lethal DNA Damage. Mol. Cancer Ther..

[133] Choul-li S., Drobecq H., Aumercier M. (2009). DNA-Dependent Protein Kinase Is a Novel Interaction Partner for Ets-1 Isoforms. Biochem. Biophys. Res. Commun..

[134] Hussain M., Daignault-Newton S., Twardowski P.W., Albany C., Stein M.N., Kunju L.P., Siddiqui J., Wu Y.-M., Robinson D., Lonigro R.J. (2018). Targeting Androgen Receptor and DNA Repair in Metastatic Castration-Resistant Prostate Cancer: Results From NCI 9012. J. Clin. Oncol..

